# The Interrelation of Synthesis Conditions and Wettability Properties of the Porous Anodic Alumina Membranes

**DOI:** 10.3390/nano12142382

**Published:** 2022-07-12

**Authors:** Daria I. Tishkevich, Alla I. Vorobjova, Anastasia A. Bondaruk, Elena S. Dashkevich, Dmitry L. Shimanovich, Ihar U. Razanau, Tatiana I. Zubar, Dmitry V. Yakimchuk, Mengge G. Dong, M. I. Sayyed, Hamoud H. Somaily, Denis A. Vinnik, Maxim V. Silibin, Sergei V. Trukhanov, Valery M. Fedosyuk, Alex V. Trukhanov

**Affiliations:** 1 Laboratory of Magnetic Film Physics, Laboratory of Physical and Chemical Technologies, Cryogenic Research Department, Scientific-Practical Materials Research Centre of NAS of Belarus, 220072 Minsk, Belarus; bondaruk625@gmail.com (A.A.B.); gramovich5@yandex.ru (E.S.D.); ir23.by@gmail.com (I.U.R.); fix.tatyana@gmail.com (T.I.Z.); dim2yakim@gmail.com (D.V.Y.); sv_truhanov@mail.ru (S.V.T.); fedosyuk@physics.by (V.M.F.); truhanov86@mail.ru (A.V.T.); 2 Laboratory of Single Crystal Growth, South Ural State University, 454080 Chelyabinsk, Russia; denisvinnik@gmail.com; 3 Micro- and Nanoelectronics Department, Belarusian State University of Informatics and Radioelectronics, 220013 Minsk, Belarus; vorobjova@bsuir.by (A.I.V.); shdl@tut.by (D.L.S.); 4 Department of Resource and Environment, Northeastern University, Shenyang 110819, China; mg_dong@163.com; 5 Department of Physics, Faculty of Science, Isra University, Amman 1162, Jordan; dr.mabualssayed@gmail.com; 6 Research Center for Advanced Materials Science (RCAMS), King Khalid University, P.O. Box 9004, Abha 61413, Saudi Arabia; hhsamily@kku.edu.sa; 7 Department of Physics, Faculty of Science, King Khalid University, P.O. Box 9004, Abha 61413, Saudi Arabia; 8 Scientific and Technological Park of Biomedicine, I.M. Sechenov First Moscow State Medical University, 119991 Moscow, Russia; sil_m@mail.ru; 9 Smart Sensors Laboratory, National University of Science and Technology MISiS, 119049 Moscow, Russia

**Keywords:** anodization, porous anodic alumina, membrane, wettability, hydrophilicity, contact angle

## Abstract

The results of studies on the wettability properties and preparation of porous anodic alumina (PAA) membranes with a 3.3 ± 0.2 μm thickness and a variety of pore sizes are presented in this article. The wettability feature results, as well as the fabrication processing characteristics and morphology, are presented. The microstructure effect of these surfaces on wettability properties is analyzed in comparison to outer PAA surfaces. The interfacial contact angle was measured for amorphous PAA membranes as-fabricated and after a modification technique (pore widening), with pore sizes ranging from 20 to 130 nm. Different surface morphologies of such alumina can be obtained by adjusting synthesis conditions, which allows the surface properties to change from hydrophilic (contact angle is approximately 13°) to hydrophobic (contact angle is 100°). This research could propose a new method for designing functional surfaces with tunable wettability. The potential applications of ordinary alumina as multifunctional films are demonstrated.

## 1. Introduction

Scientists are continually attracted to porous anodic alumina because of its unique ordered honeycomb cell structure. Such a structure can be used to make a variety of new multifunctional films, micro- and nanoelements utilizing the template-assistant approach. [1,2,3,4,5,6,7,8]. Lately, PAA has been intensely investigated for applications in medical and biotechnological development, including drug delivery systems and biosensor devices, bio-filtration for the separation of proteins and organic molecules, and as a template for biocompatible tissue preparation, etc. [9,10,11]. It is essential for medical applications that the PAA pores have controllable sizes ranging from several to hundreds of nanometers, which are comparable to the sizes of intracellular biological objects. Aluminum is electrochemically anodized to produce a nanoporous multichannel alumina structure with pore sizes ranging from 10 to 150 nm and a density of almost 1 × 10^10^ pores per cm^2^ [12,13,14]. This method is less difficult than traditional lithographic techniques. In this circumstance, modifying the PAA surface to achieve pore walls and a well-wetted surface is preferable.

One of the most important tasks from a practical point of view is protein and organic macromolecule separations [10]. The use of membranes with a regular porous structure and uniformly sized cylindrical pores was proposed to increase the productivity and selectivity of the membranes in the separation of biomolecules [10,15]. Further selectivity in bioseparation systems can be achieved by optimizing such characteristics as pore walls, modifications of the PAA surface itself, pore diameter, and length [16,17].

Another area of active use of PAA membranes is UV-nanoimprint lithography (NIL). The fabrication of a large-area, high-resolution stamp (matrix) for nanoimprinting is a key step in UV-NIL. The use of a PAA membrane as a primary matrix for the manufacture of a soft mold replica for a large area of UV-NIL has been proposed [18]. In this case, the PAA membrane must have close to hydrophobic properties in order to separate the press mold without damage.

The main factor for obtaining a superhydrophobic PAA surface is high surface roughness in the form of hierarchical micro- or nanoscale structures combined with the material’s low surface energy [19,20,21]. Such surfaces, which have a high interfacial contact angle with water and low adhesion, are attracting much more attention because of their variety of applications in functional microfluidic devices.

Scientific study into the development of non-wettable and/or self-cleaning surfaces was motivated by some natural materials’ superhydrophobicity and capacity to remove surface contamination. According to the Cassie–Baxter theory, high surface roughness and low surface energy of the material are required for the practical application of such a surface (i.e., high values of the contact angle). In this regard, research into the fabrication of such coatings is performed in two directions: changing the surface of hydrophobic materials and coating with a rough surface using a material with low surface energy. The various surface treatment options include template methods, chemical, plasma-chemical, or ion etching methods, electrochemical methods (anodizing and deposition), surface nanostructuring (creating arrays of nanopillars or nanowires), etc. The methods for changing the surface morphology include these options. The latter option is described in the literature by techniques for producing arrays of nanostructures using different materials, such as TiO_2_-based nanostructured surfaces [22,23,24,25,26,27] or morphological modification of the polymer surface by replicating the anodic alumina structure [28,29].

High quality crystalline Al_2_O_3_ is extensively used as the promising diagnostics/window materials for deuterium–tritium fusion reactors due to its high resistance to rapid neutrons and capacity to preserve mechanical and electrical integrity [30,31,32]. For instance, highly sensitive luminescent ionizing radiation detectors are built on specifically treated alumina [33,34]. The optical characteristics of the alumina, which depend on the oxide quality, namely on point defects and the PAA surface defects, are significant in this situation [35,36].

By regulating the synthesis conditions, various surface morphologies of these PAA films can be obtained. Such alumina surfaces without a special (usually organic) coating of materials with low energy demonstrate notable morphology-dependent wettability. In this case, the PAA surface consists of only the porous layer modified in the manufacturing process after a pore widening operation (etching of a barrier layer).

Many research groups study the wettability properties of the PAA, but mainly after a special pre-treatment of the PAA (after changing the surface chemistry of the PAA nanostructure) [37,38,39,40]. There are also works [41,42] that perform the effect of various alumina formation conditions on wettability. For example, [41] shows the effect of pore-widening duration on the wetting properties of nanostructured PAA membranes fabricated in electrolytes based on sulphuric and oxalic acid. In [40], reports about different surface morphologies of nanostructured alumina films obtained under high-field anodization in oxalic acid electrolytes. In [42], the influence of duration of the second step of anodization, electrolyte type, and substrate purity on the state of the PAA surface is performed.

In principle, wetting behavior can be changed from the Wenzel’s model (which describes the homogeneous wetting regime) to the Cassie–Baxter model (which describes the heterogonous wetting mode, which is more complex) by changing only the surface morphology [40,43,44]. In particular, an artificially unmodified PAA surface, consisting of a porous layer and hierarchical nanostructures in the form of, for example, nanowires, nanopillars, or pyramids, etc., demonstrates both superhydrophobicity and superoleophilicity, which can be used in water-proof or self-cleaning surfaces, functional microfluidics devices, and water separation, etc. The aim of this work is to propose a new approach for the design of functional surfaces with tunable wettability properties.

Our group has traditionally been involved in electrochemical anodizing and deposition processes, so we are conducting research on the creation of nanostructured materials using these methods. Anodic alumina is used as a template for creating arrays of various nanostructures: nanopillars, nanowires, nanotubes. The advantages of alumina are quite widely described in our previous works. In this case the following can be noted: sufficiently high mechanical strength and thermal conductivity [45], high electrochemical resistance [13], resistant to the high temperatures [46]. Moreover, as already mentioned, its geometric characteristics can be easily adjusted by synthesis conditions’ changing [47,48]. Such templates are considered as the most relevant for integration into microelectronic technologies [1,49,50,51].

In a preceding study [29], we represented the results of the fabrication and investigation of the wetting properties of PAA with a thickness of 25 to 75 m and various pore sizes. The effect of morphology on the wetting properties is shown through a comparison of the outer and inner PAA surfaces. The wettability of both alumina surfaces is morphology-dependent. Our systematic studies of the PAA surface properties have shown that the contact angle is a very sensitive parameter and its value is determined by a variety of parameters, including surface pretreatment (modification due to pore widening) during the manufacturing process of PAA of different thicknesses for different purposes. However, it is possible to obtain quite reliable and reproducible results with the constancy of the determining technological factors.

In this work, we would like to demonstrate how the PAA outer surface properties change in the process of production. The interfacial contact angle was measured to investigate the wettability properties of the outer PAA surface. We want to illustrate that the PAA wettability changes during the production process, and this must be put into consideration in order to get a template with reproducible parameters that can be used for a variety of membrane applications. Consequently, PAA samples with various morphological characteristics were obtained, and their properties of wettability were studied.

## 2. Materials and Methods

### 2.1. Materials

**Figure 1:**
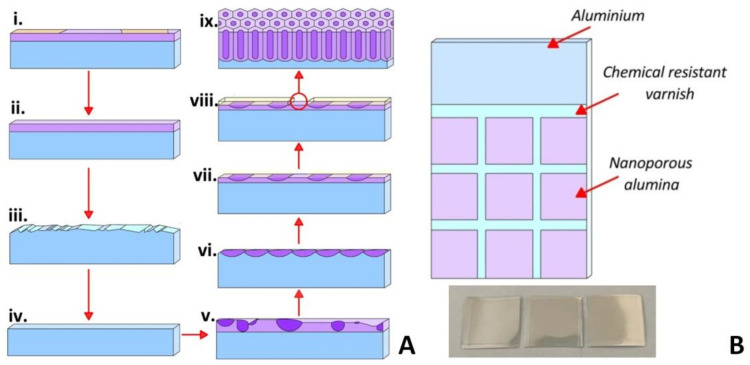
Scheme of the PAA fabrication process using two-step anodization for an ordered PAA formation (**A**); schematic view and Al substrate image for the PAA membranes obtaining (**B**).

The as-fabricated PAA membranes were developed in this study by one-step (I type) and two-step (II type) anodizing processes in a 4% C_2_H_2_O_4_ water-based solution at 15 °C. A high purity aluminum foil (100 μm thick, 99.9994% purity) was used as an initial material. Figure 1A shows the scheme of the PA fabrication process using two-step anodization.

The process of an ordered porous PAA matrix formation is two-step anodization and includes the following technological steps: (i.) fabrication of the substrates with the required geometrical dimensions (Figure 1B) from Al foil; (ii.) substrates’ washing in a H_2_SO_4_ + CrO_3_ solution for 10 min; (iii.) chemical etching of the substrate surface in 10% NaOH solution at 40 °C for 3 min; (iv.) chemical polishing of the Al substrates in H_3_PO_4_ + HNO_3_ solution at 80 °C for 1.5 min; (v.) first-stage anodizing in 4% C_2_H_2_O_4_ at 40, 50, 60 V for 60 min at a temperature of 15 °C); (vi.) etching of the PAA formed during the first-stage anodization in a solution of H_2_O + CrO_3_ + H_3_PO_4_ at 80 °C for 1–5 min; (vii.) formation of PAA thin adhesive oxide for the chemical resistant varnishing (CRV), inset in Figure 1B: 4% C_2_H_2_O_4_ at voltages: 40, 50, and 60 V for 30 s; (viii.) CRV of the samples with an air drying for at least 12 h; (ix.) second-stage anodizing in 4% C_2_H_2_O_4_ at 40, 50, and 60 V for 30 min at a temperature of 15 °C; (x.) mechanical removal of the CRV and cutting of substrates into individual modules for further studies of the microstructure and surface wettability. All chemicals were of analytical grade. One-stage anodizing was carried out at voltages of 40, 50, and 60 V for 30 min at 15 °C.

PAA’s outer surface was chemically altered for 5, 10, 15, and 20 min at (35 ± 2) °C using a water-based solution of 5% H_3_PO_4_. For the obtaining of PAA membranes, chemical etching of a barrier layer at the pore bottom is usually carried out, that is, PAA with through pores. This process is isotropic. Therefore, the pore walls of the oxide cells are simultaneously etched (chemical pore widening), that is, the pore diameter changes (increases). The PAA membrane production is described in more detail in [52].

**Figure 2:**
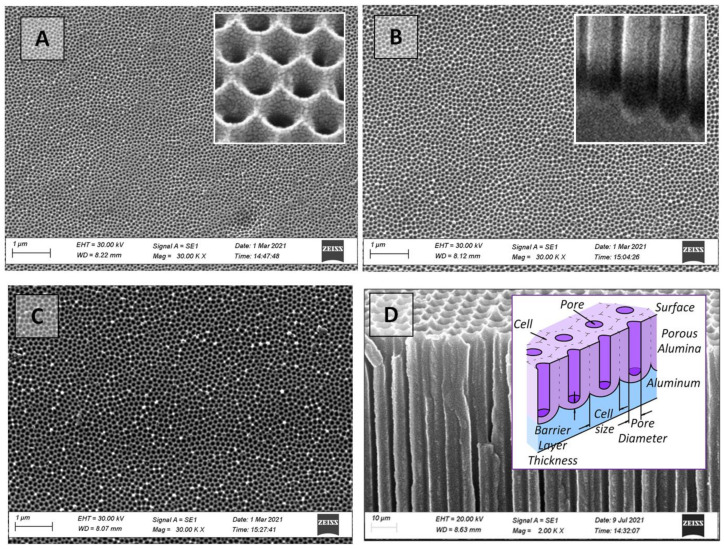
SEM images of the top view (outer surface) of as-fabricated PAA membranes with a size of 15 mm × 15 mm: (**A**)—40 V, (**B**)—50 V, (**C**)—60 V, (**D**)—PAA cross-section. The insets show enlarged images of the surface (**A**) and the bottom part of the PAA cross-section (**B**); and a schematic cross-section view of the PAA (**D**).

As a consequence, PAA membranes with a well-ordered structure and 3 µm (40 V), 3.3 µm (50 V), and 3.6 µm (60 V) thicknesses with different pore diameters have been obtained (Figure 2). It was shown in [41,53] that when the thickness of as-fabricated PAA is less than 1.7 µm, the surface morphology is the only significant factor determining the value of the contact angle, which was also verified by our experimental results for as-fabricated PAA with a small thickness of 3–3.6 µm.

### 2.2. Methods

The PAA sample morphology (cell diameter *D* or interpore distance, thickness *H*, and pore diameter *d*) was investigated via Carl Ziess EVO10 (Carl Ziess, Oberkochen, Germany) scanning electron microscopy (SEM). The experimental data was processed using the software package “SmartSEM” (Carl Ziess, Oberkochen, Germany). The graphic editor “Image J” (Wayne Rasband (NIH), Kensington, MD, USA) was also used to examine the SEM pictures. The conventional technique described in detail in [54,55] was used to perform the pore size statistical analysis. For at least three SEM pictures, pore sizes were measured using the software “SmartSEM.” The porosity was estimated using custom software that analyzed the SEM images and then provided porosity contrast images.

The following formula was used for the porosity value estimation [56]:

(1) $$\alpha =\frac{S_{ppa}}{S_{gpa}}\cdot 100\%$$

where *S_ppa_*—the pores projection area and *S_gpa_*—is the grains projection area.

The following equations for *S_ppa_* and *S_gpa_* can be further advanced by assuming that each single pore is a perfect circle [56]:

*S_ppa_* = *π* × (*d*/2)^2^ (2)

(3) $$S_{\mathrm{gpa}}=\frac{\surd 3\times D^{2}}{2}$$

When Equations (2) and (3) are substituted into Equation (1), the following equation for the porosity of a hexagonally organized cell nanostructure is obtained:

(4) $$\alpha =\frac{\pi }{2\sqrt{3}}{\left(\frac{d}{D}\right)}^{2}=0.907\;{\left(\frac{d}{D}\right)}^{2}$$

Both Equations (1) and (4) were used in the research.

The “recumbent drop” method was used to determine the wettability properties of PAA by measuring the interfacial contact angle (ICA—*θ*) [57]. From the Thermo Scientific Finnpipette microdoser (ThermoFisher Scientific, Waltham, MA, USA), a drop of distilled water of the order of 15 μL was applied to the PAA surface. The ICA was defined using the drop’s basic dimensions and the condition that *θ* < 90° according to the following formula:

(5) $$tg\Theta =\frac{2hr}{r^{2}-h^{2}}$$

where:

*θ*—the ICA,
*r*—the radius of the drop contact area with the PAA surface,
*h*—the height of the drop.

The contact angle was measured after 1, 2, and 5 min when the drop was applied. Three measurements were made for each of the three drops, and the average was used.

## 3. Results and Discussion

### 3.1. Topological features of the PAA

The obtained samples were analyzed using the SEM technique after each main stage of the PAA production process. Chemical etching technology [58] was used to remove the barrier layer at the bottom of the pores, and several types of samples with different pore sizes were produced (Table 1).

With a proportionality constant *λ_p_* of approximately 1.29 nm × V^−1^, the pore diameter of the PAA *d* is linearly proportional to the anodizing voltage *U* [59]:

*d* = *λ_p_* × *U* (6)

where:

*d*—a diameter of pore (nm),
*U*—an anodizing voltage (V).

The diameter–voltage correlation is not susceptible to the electrolyte type and does not alter considerably with anodizing time [60]. At steady-state growth of PAA, the interpore distance (cell diameter) of PAA *D* is directly proportional to the anodizing voltage, with a proportionality constant *λ_c_* of almost 2.5 nm × V^−1^ [61]:

*D* = λ*_c_* × *U* (7)

The other significant microstructure parameters of the PAA, such as the cell wall thickness (*W*) and the barrier layer thicknesses (*B*) are presented in Table 1. The cell wall thickness *W* of densely packed, highly ordered hexagonal cells of PAA membranes with a diameter *D* can be defined using the Formula (8) and is equal to 32.5 nm [56]:

(8) $$W=\frac{D-d}{2}$$

Additionally, the wall thickness is correlated with the barrier layer thickness as the following ratio and is equal to 36.4 nm [62]:

(9) B=1.12·W

Moreover, the pore density, *P_p_*, was calculated by equation *P_p =_* $\frac{2}{\sqrt{3}D^{2}}$ [31] to be approximately 10^10^ per cm^2^: 1.16 × 10^10^ (40 V); 0.7 × 10^10^ (50 V); 0.45 × 10^10^ (60 V).

**Figure 3:**
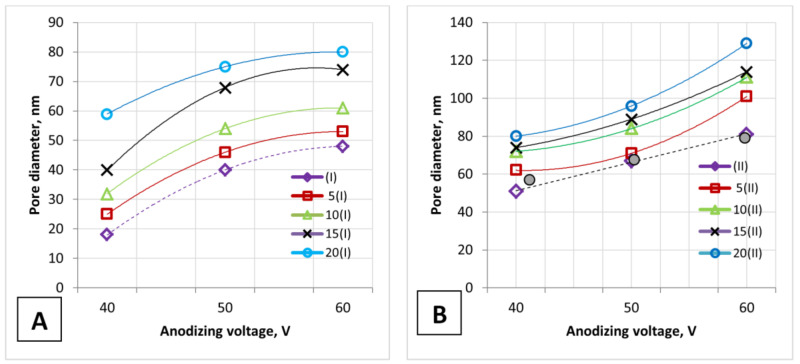
The anodizing voltage influence on the PAA pore diameter: (**A**)—one-stage anodizing and (**B**)—two-stage anodizing. The calculated values are shown as shaded circles.

**Figure 4:**
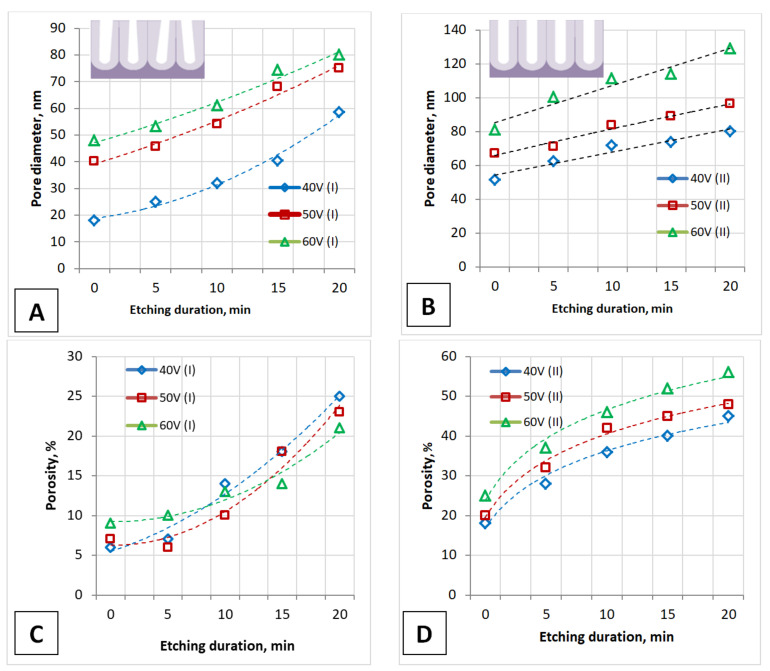
The influence of the main parameters of PAA obtaining (anodizing voltage and etching duration of the barrier layer) on the pore diameter of PAA during one-stage (**A**) and two-stage (**B**) anodizing and on the PAA porosity during one-stage (**C**) and two-stage (**D**) anodizing. The insets schematically show the cross-section of one-stage (**A**,**C**) and two-stage (**B**,**D**) anodized PAA fragments.

**Figure 5:**
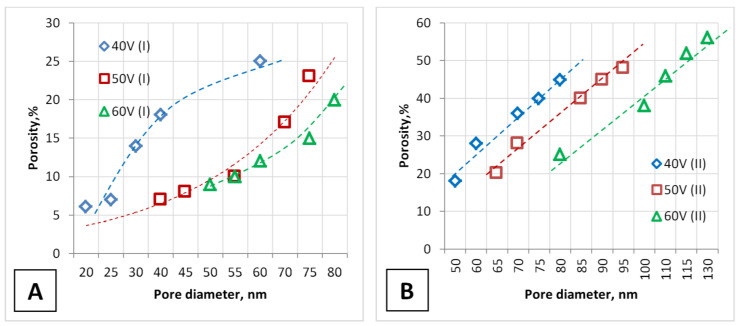
Dependence of the PAA porosity during one-stage (**A**) and two-stage (**B**) anodizing on the pore diameter and voltage.

It should be noted that the calculated values of all the presented morphological parameters for the I type samples (for PAA formed by one-stage anodizing) (Table 1) differ from the experimental data and almost correspond to the data for PAA formed by two-stage anodizing. This is also seen from the dependencies presented in Figure 3, Figure 4 and Figure 5 and explains the difference in experimental data from various literature sources [1,2,3,48,63].

This is due to the fact that Formulas (6)–(9) were determined for an ideal PAA with a hexagonally ordered structure. Such PA can be produced using an imprinting method [64,65] or two-stage anodization [66,67]. Therefore, for samples of the I type formed by one-stage anodizing, the experimental values of the parameters differ significantly from the calculated ones. It is practically impossible to obtain a good-ordered PAA during the 30 min of one-stage anodizing. For samples of the II type, obtained by two-stage anodization, the porosity values are slightly different, which is obviously due to the method of measuring porosity by the pore diameter on the PAA outer surface. Similar differences have been observed in other works [41,68].

Figure 3 depicts the PAA pore diameter (on the outer surface of the PAA) versus anodizing voltage for an unetched PAA surface (dashed lines) and an etched PAA surface (solid lines).

The calculated values (shaded circles, Figure 3B) almost correspond to the experimental values for an unetched PAA surface (as-fabricated sample, before etching) for the II type of samples. Only the experimentally obtained pore diameter of the I type samples (Samples No. 1, 2, 3) and the experimental porosity of the II type samples (Samples No. 4 and 6) differ from the calculated values. This is due to the fact that the calculation equations are obtained for densely packed, highly arranged, hexagonal PAA cells, and each solitary pore is assumed to be a perfect circle. Figure 4 presents the influence of the main parameters of PAA on the pore diameter and porosity of the PAA during one- and two-stage anodizing.

Figure 5 shows the dependence of PAA porosity versus pore diameter for both types of samples.

**Figure 6:**
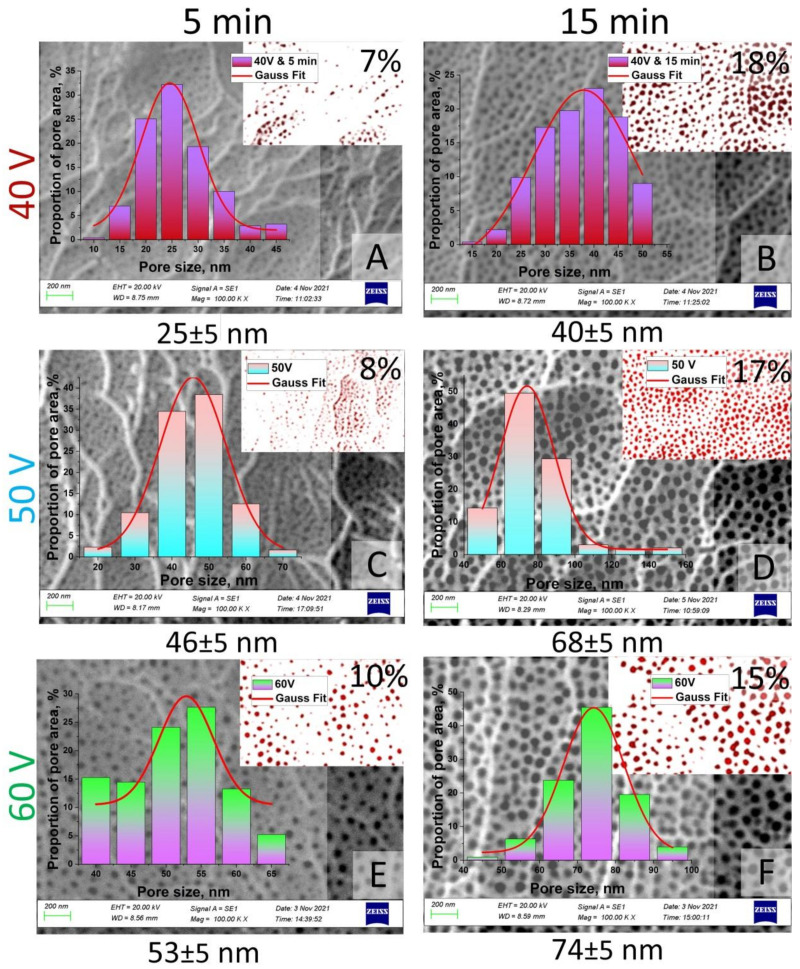
SEM images (contrast SEM images of porosity—red and white) and pore size histograms of the I type samples after chemical modification: (**A**)—Sample No. 1 after chemical etching during 5 min; (**B**)—Sample No. 1 after chemical etching during 15 min; (**C**)—Sample No. 2 after chemical etching during 5 min; (**D**)—Sample No. 2 after chemical etching during 15 min; (**E**)—Sample No. 3 after chemical etching during 5 min; (**F**)—Sample No. 3 after chemical etching during 15 min.

**Figure 7:**
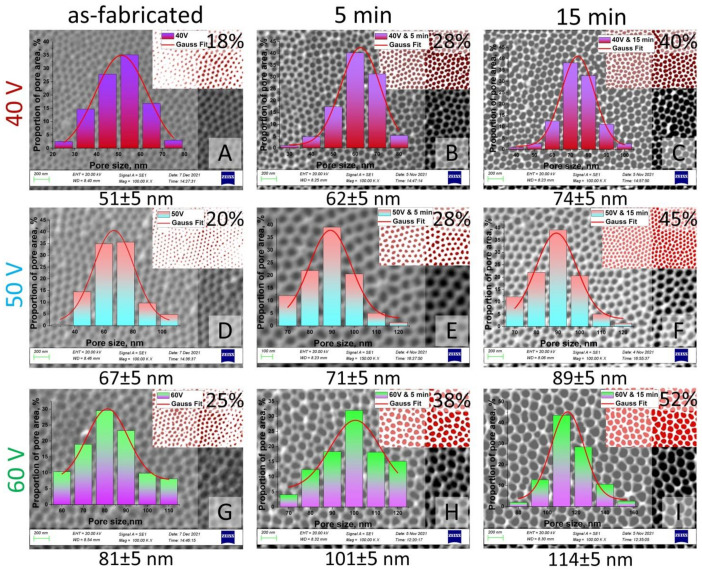
SEM images (contrast SEM images of porosity—red and white) and pore size histograms of the II type samples after chemical modification: (**A**)—Sample No. 4 as-fabricated; (**B**)—Sample No. 4 after chemical etching during 5 min; (**C**)—Sample No. 4 after chemical etching during 15 min; (**D**)—Sample No. 5 as-fabricated; (**E**)—Sample No. 5 after chemical etching during 5 min; (**F**)—Sample No. 5 after chemical etching during 15 min; (**G**)—Sample No. 6 as-fabricated; (**H**)—Sample No. 6 after chemical etching during 5 min; (**I**)—Sample No. 6 after chemical etching during 15 min.

The pore size, diameter, length, and porosity were determined using SmartSEM software. Figure 6 and Figure 7 represent the results of statistical analysis of the main morphological parameters of the samples for both types of samples: SEM images, porosity, bar graph, and contrast images. Inserts (red with white) in Figure 6 and Figure 7 show the contrast SEM images of porosity (in %).

For both types of samples obtained at one-stage and two-stage anodizing, the pore diameter is proportional to the anodizing voltage. Moreover, the pore diameter is dependent on the duration of the barrier layer etching and is almost 1.5–1.7 times greater in the case of the two-stage anodization. Porosity also depends on the anodizing voltage for both types of samples and is proportional to the pore diameter at a given voltage. The porosity of PAA (I type) is also less than that of PAA (II type) since the pore diameter of these PAAs is different.

It can be concluded that such an important parameter of the PAA as porosity depends on the channel structure and pore diameter (and, therefore, penetrating ability) [69].

The barrier layer was etched in all samples of both types by immersing the entire PAA in a 5% H_3_PO_4_ water-based solution for 5, 10, 15, and 20 min at a temperature of (35 ± 2) °C.

In general, SEM pictures and size histograms (Figure 6 and Figure 7) of PAA samples produced in the optimal etching mode indicate that the PAA surface morphological characteristics are relatively uniform.

### 3.2. The Wetting Properties of PAA

**Figure 8:**
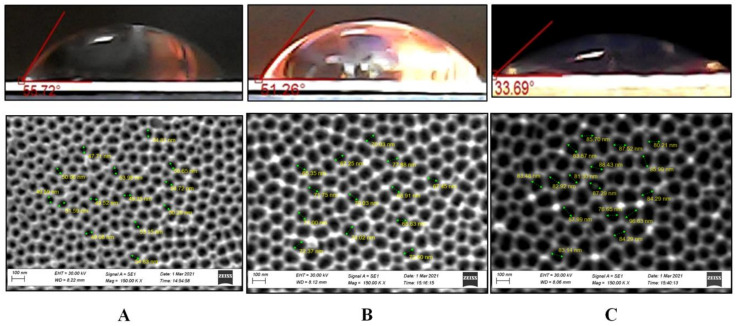
Contact angle as a surface topology function for as-fabricated samples No. 4 (**A**), 5 (**B**), and 6 (**C**) of the II type and corresponding SEM images of outer surfaces.

**Figure 9:**
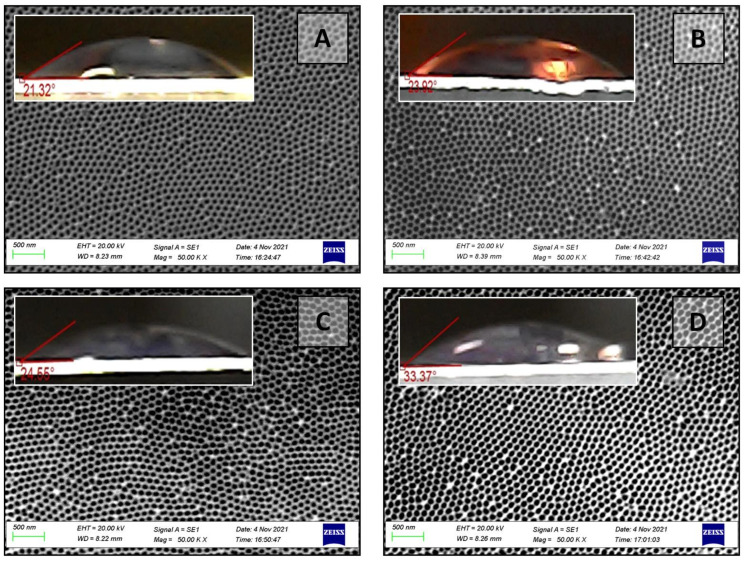
Contact angle as a surface topology function of the PAA sample No.5 of the II type after chemical modification in 5% H3PO4 for 5 min (**A**), 10 min (**B**), 15 min (**C**), 20 min (**D**), and corresponding outer surface SEM images.

The topological properties of the PAA surface can vary significantly depending on the through-pores obtaining mechanism, as shown by these results and previously provided data [14]. As a result, the contact angle on the outer side of various types of PAA was evaluated before and after chemical modification. Figure 8 demonstrates the contact angle and SEM images of the outer surface of as-fabricated samples No. 1, 2, and 3 of the II type. Figure 9 depicts the contact angle and SEM images of the outer surfaces of a PAA sample No. 5 of the II type after pores opened due to different durations of the barrier layer chemical etching.

**Figure 10:**
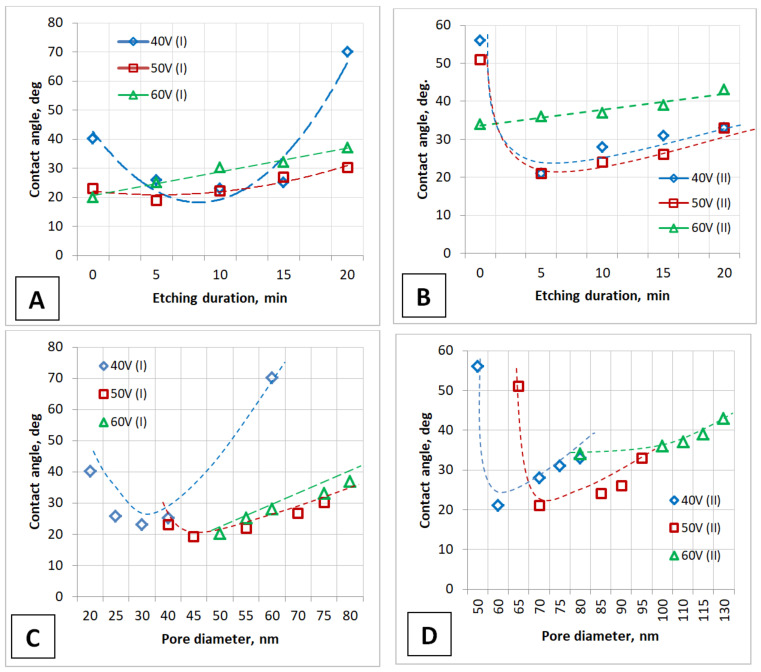
The dependence of the contact angle on the PAA pore diameter and etching duration of the barrier layer obtained during one-stage (**A**,**C**) and two-stage (**B**,**D**) anodizing after chemical modification (after 1 min the water drop applying).

All PAA samples after etching obtained similar results. The results as contact angle versus etch time and pore diameter are presented in Figure 10 and Table 2.

All samples have a contact angle of less than 90°, which indicates the hydrophilicity of the PAA surface. It can be seen from the presented results that the contact angle values depend on both the anodizing voltage (i.e., the distance between the pores) and the etching time (i.e., the pore diameter). The nature of the dependences is similar for samples of both types and is ultimately determined by the pore diameter, which is especially clearly seen from a comparison of the contact angle values for two types of samples: they are approximately the same and lie in the range approximately from 20 to 40° after 1 min of water drop application. This dependence is more pronounced for PAA obtained at lower anodizing voltages.

**Figure 11:**
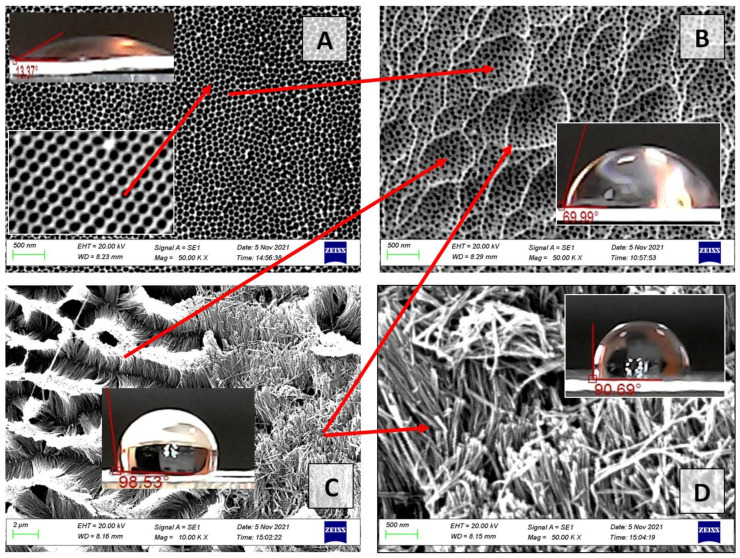
The SEM images of the outer surfaces (surface topology) for the PAA sample No. 4 of the II type: (**A**)—sample No. 4 (40 V, type II) after 15 min of chemical etching in 5% H_3_PO_4_; (**B**)—sample No. 1 (40 V, type I) after 15 min of chemical etching in 5% H_3_PO_4_ during; (**C**,**D**)—sample No. 4 after 20 min of chemical etching in 5% H_3_PO_4_.

This is due to the fact that during the barrier layer chemical etching, not only the pore bottoms, but also the pore walls (oxide cells) are etched. For example, for sample No. 1 of the II type (40 V), after 20 min of etching, the pore walls become so thin that the oxide cells begin to gather into bundles or nanowire pyramids (Figure 11).

The drops do not spread well on such a surface. By the way, such a variant of the PAA surface is described in [40,42,61] for obtaining a superhydrophobic surface. Because of the wide range of applications, notably in medicine, the fabrication of such PAA (smart nanomaterials) is a popular research area. [70,71].

**Figure 12:**
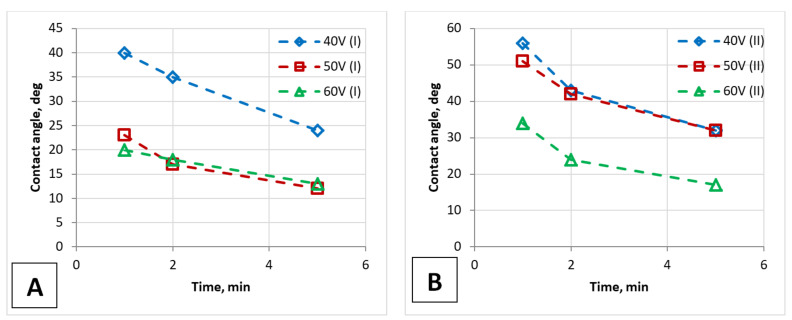
Demonstration of a PAA film’s surface wetting ability (contact angle): the graph for contact angles during the applying of a water drop on the PAA surfaces of the one-stage (**A**) and two-stage (**B**) anodized samples.

Figure 11 shows that nanowires (NWs) from the walls of PAA cells within one ordered region (shown by red arrows in Figure 11C) are joined (electrostatically attracted to each other) and pyramidal structures of the PAA NWs are formed at the sites of PAA cells within one ordered area. That is, within each ordered region of PA (15 min etching), the hexagonal oxide cells form an ordered structure, a magnified image of which is shown in the inset in Figure 11A. After 20 min of etching, these cells close within each area and form a new hierarchical structure with an architecture depending on the initial PA morphology (pore diameter, distance between them, pore height). If the pore height (oxide thickness) is small, the barrier layer etching rate at the pore bottoms and on the pore walls is approximately the same. The concentration and temperature of the etching solution along the nanopore channels are almost homogeneous. However, at the boundaries of ordered regions, PAA cells dissolve faster than within the same ordered regions because of defect emergence or distortion of the side walls of pores (internal mechanical stresses), which causes the formation of PAA NWs. At the cell boundaries, the PAA NWs do not close and are located vertically (Figure 11C,D). The insets show how the contact angle changes on different types of surfaces of one sample during its fabrication. On the initial surface of the ordered PAA, sample No. 4 (40 V, type II), the contact angle corresponds to a superhydrophilic surface and is equal to 13.3 ± 2°. On the pyramidal surface and on the NWs surface, the contact angle increases and approaches the values for the superhydrophobic surface (90 and 100°). If a drop of water is placed on such a surface, it retains a spherical shape for a long time and does not spread over the surface of the sample. That is, we obtain a hydrophobic surface without pretreatment of the PAA surface with organic reagents, which exhibits a stable character in air, and the contact angle does not show visible changes in shape. On other samples, we measured the contact angle depending on the time of application to the surface of the PAA (Figure 12).

It is clear from the provided results that the contact angle values depend not only on the morphology of the PAA surface but also on the time the drop applies to the sample surface, i.e., on the measurement technique. Therefore, the ability of PAA film to absorb humidity was investigated by measuring the contact angles depending on the time of applying a water drop to the surface. It can be seen from these dependences that the contact angle also depends on the time after which it was measured, that is, on the time the drop was on the surface (Figure 12). The same dependence was observed in the work [41].

These measurements of the contact angles of the time-dependent deposition of water drops on the PAA film made it possible to: i. illustrate the possibility of obtaining a sufficiently effective anti-drop coating on samples of two types; ii. determine that the wetting process is stabilized after two minutes of applying; iii. the nature of these dependences is almost identical for both types of samples.

The infiltration of water into the PAA pores causes the contact angle in both cases to decrease after a few minutes. The mechanism PAA wetting has been clarified as follows [72]: if the water can diffuse along the walls of the PAA pores, then the liquid is first brought into contact with the PAA surface and then the low-energy liquid rapidly distributes to the surfaces of high-energy. The short-range and long-range polar interactions between the wetting liquid and the pore walls are the driving forces in this mechanism [72].

According to the results obtained, the contact angle is dependent on a PAA characteristic such as pore diameter (porosity). The porosity is determined by such technological parameters as the etching duration of the barrier layer and the anodizing voltage. Furthermore, the dependence on the pore diameter is extreme for 40 and 50 V anodizing voltage. As expected, the contact angle increases in proportion to the PAA pore diameter (porosity).

Moreover, the contact angle values and the physicochemical properties of the PAA surface, also known as “surface chemistry”, have a significant impact. We do not modify this factor within this article to specify that the contact angle is influenced by the intrinsic features of the as-fabricated amorphous membrane. This question has no data in the research literature. Such studies are planned for the next article.

Next, we are going to produce and examine a simple technology for designing such a surface (architecture) of alumina nanowires array, based on which it will be possible to obtain surfaces with improved properties. In order to accomplish this, we intend to apply surface modification to the obtained samples to increase the contact angle and obtain surfaces with a high specific surface area and capacity for self-cleaning, with a focus on the bioinspired design of surfaces with micro-droplet self-assembly.

## 4. Conclusions

It has been reported that PAA membranes can be fabricated by two-step anodization of aluminum foil in an oxalic acid electrolyte. PAA membranes with pore diameters ranging from 25 to 130 nm and a thickness of 3.3 ± 0.2 µm were developed. At 40, 50, and 60 V, anodization in 4% oxalic acid was performed. In order to investigate solely the effect of the PAA surface morphology on the wettability properties, the surface chemistry of the PAA samples was not changed. The contact angle was demonstrated to be dependent not only on the pore diameter and anodization voltage but also on the duration of the barrier layer etching, indicating that it changes throughout the production process. The pore diameter and contact angle almost linearly increase with the rise in etching duration, but the nature of this process depends on the type of anodizing. The contact angle values depend not only on the morphology of the PAA surface but also on the time the drop applies to the sample surface, i.e., on the measurement technique. Different surface morphologies of such PAA membranes can be obtained by adjusting synthesis conditions. This allows the surface properties from hydrophilic to hydrophobic to change without the surface chemistry of the PAA samples (without pretreatment of the PAA surface with organic reagents) being changed. The self-assembled hierarchical surfaces of the PAA exhibit an anti-adhesion to water and exhibit water-repellent properties. This water-repellent behavior could find practical applications such as drag-reduction and self-cleaning coatings. The obtained results demonstrate that, depending on the future application of PAA membranes, the appropriate PAA production conditions (substrate purity, anodizing voltage, duration of the second anodizing step, especially etching duration) can be selected.

## Figures and Tables

**Table 1:** The experimental samples’ primary morphological features (before etching, as-fabricated amorphous PAA).

Sample Type	Sample No.	Anodizing Voltage *U*, V	Pore Diameter *d*, nm	Cell Diameter *D*, nm	Porosity *α, %*	Barrier Layer Thickness *B,* nm	Wall Thickness W, nm	d/D
I	1	40	(22 ± 5)	(100 ± 7)	(6)	44	39	0.18
I	2	50	(40 ± 5)	(120 ± 7)	(7)	34	30	0.33
I	3	60	(48 ± 5)	(140 ± 5)	(9)	52	46	0.34
II	4	40	51.6* (50 ± 5)	100 * (105 ± 5)	24.6 * (18)	27.1 * (30)	24.2 * (27)	0.52 * (0.49)
II	5	50	64.5 * (67 ± 5)	125 * (130 ± 5)	24.6 * (20)	33.9 * (35)	30.3 * (32)	0.52 * (0.52)
II	6	60	77.4 * (81 ± 5)	150 * (160 ± 5)	24.6 * (25)	40.7 * (44)	36.3 * (39)	0.52 * (0.51)

* The calculated and mean values were received using SEM images (in brackets).

**Table 2:** The main conditions for PAA preparation of the experimental samples (as-fabricated and after chemical modification) and the main parameters of both types of samples.

Sample Type	Sample No.	Anodizing Voltage *U*, V	Etching Duration, Min	Pore Diameter d, nm	Porosity *α, %*	Contact Angle, Deg.
I	1	40	0	18 ± 5	6 ± 2	40.4 ± 2
I	1	40	5	25 ± 5	7 ± 2	25.7 ± 2
I	1	40	10	32 ± 5	14 ± 2	23.1 ± 2
I	1	40	15	40 ± 5	18 ± 2	25.0 ± 2
I	1	40	20	59 ± 5	25 ± 2	69.9 ± 2
I	2	50	0	40 ± 5	7 ± 2	23.0 ± 2
I	2	50	5	46 ± 5	8 ± 2	19.2 ± 2
I	2	50	10	54 ± 5	10 ± 2	21.8 ± 2
I	2	50	15	68 ± 5	17 ± 2	26.7 ± 2
I	2	50	20	75 ± 5	23 ± 2	29.8 ± 2
I	3	60	0	48 ± 5	9 ± 2	20.4 ± 2
I	3	60	5	53 ± 5	10 ± 2	25.0 ± 2
I	3	60	10	61 ± 5	12 ± 2	29.6 ± 2
I	3	60	15	74 ± 5	15 ± 2	31.7 ± 2
I	3	60	20	80 ± 5	21 ± 2	36.4 ± 2
II	4	40	0	51 ± 5	18 ± 2	55.7 ± 2
II	4	40	5	62 ± 5	28 ± 2	20.6 ± 2
II	4	40	10	72 ± 5	36 ± 2	27.6 ± 2
II	4	40	15	74 ± 5	40 ± 2	30.9 ± 2
II	4	40	20	80 ± 5	45 ± 2	32.5 ± 2
II	5	50	0	67 ± 5	20 ± 2	51.2 ± 2
II	5	50	5	71 ± 5	28 ± 2	21.3 ± 2
II	5	50	10	84 ± 5	40 ± 2	24.5 ± 2
II	5	50	15	89 ± 5	45 ± 2	24.9 ± 2
II	5	50	20	96 ± 5	48 ± 2	33.3 ± 2
II	6	60	0	81 ± 5	25 ± 2	33.6 ± 2
II	6	60	5	101 ± 5	38 ± 2	35.8 ± 2
II	6	60	10	111 ± 5	46 ± 2	36.8 ± 2
II	6	60	15	114 ± 5	52 ± 2	38.6 ± 2
II	6	60	20	129 ± 5	56 ± 2	42.8 ± 2

## Data Availability

The data presented in this study are available on request from the corresponding authors.
